# Birth weight and renal markers in children aged 5–10 years in Cameroon: a cross-sectional study

**DOI:** 10.1186/s12882-020-02133-9

**Published:** 2020-11-07

**Authors:** Francois Folefack Kaze, Seraphin Nguefack, Constantine Menkoh Asong, Jules Clement Nguedia Assob, Jobert Richie Nansseu, Mathurin Pierre Kowo, Victorine Nzana, Ginette Claude Mireille Kalla, Marie Patrice Halle

**Affiliations:** 1 Department of Internal Medicine and Specialties, Faculty of Medicine and Biomedical Sciences, University of Yaoundé 1, Yaoundé, Cameroon; 2 Departement of Paediatrics, Faculty of Medicine and Biomedical Sciences, University of Yaoundé 1, Yaoundé, Cameroon; 3 Department of Biomedical Sciences, Faculty of Health Sciences, University of Buea, Buea, Cameroon; 4 Department of Public Health, Faculty of Medicine and Biomedical Sciences, University of Yaoundé 1, Yaoundé, Cameroon; 5 Department of Clinical Sciences, Faculty of Medicine and Pharmaceutical Sciences, University of Douala, Douala, Cameroon

**Keywords:** Blood pressure, Proteinuria, Glomerular filtration rate, Children, Cameroon

## Abstract

**Background:**

A relationship exists between birth weight (BW) and glomerular filtration rate (GFR) in postnatal kidney. Willing to fill a gap of knowledge in sub-Saharan Africa, we assessed the effect of BW on blood pressure (BP), proteinuria and GFR among Cameroonians children.

**Methods:**

This was a cross-sectional hospital-based study from January to April 2018 at the Yaounde Gynaeco-Obstetric and Paediatric Hospital (YGOPH). We recruited low BW (LBW) [< 2500 g], normal BW (NBW) [2500-3999 g] and high BW (HBW) [> 4000 g] children, aged 5–10 years, born and followed-up at YGOPH. We collected socio-demographic, clinical (weight, height, BP), laboratory (proteinuria, creatinine), maternal and birth data. The estimated GFR was calculated using the Schwartz equation.

**Results:**

We included 80 children (61.2% boys) with 21 (26.2%) LBW, 45 (56.2%) NBW and 14 (15.5%) HBW; the median (interquartile range) age was 7.3 (6.3–8.1) years and 17 (21.2%) were overweight/obese. Two (2.5%) children, all with a NBW (4.4%), had an elevated BP whereas 2 (2.5%) other children, all with a LBW (9.5%), had hypertension (*p* = 0.233). Seven (8.7%) children had proteinuria with 19, 2.2 and 14.3% having LBW, NBW and HBW, respectively (*p* = 0.051). Equivalent figures were 18 (22.5%), 14.3, 24.2 and 28.6% for decreased GFR, respectively (*p* = 0.818). There was a trend towards an inverse relationship between BW and BP, proteinuria and GFR (*p* > 0.05).

**Conclusion:**

Proteinuria is more pronounced in childhood with a history of LBW and HBW while LBW children are more prone to develop hypertension. Regular follow-up is needed to implement early nephroprotective measures among children with abnormal BW.

## Background

There is a correlation between birth weight (BW) and glomerular number, density, volume, size and filtration rate in postnatal kidney [1–6]. This is supported by Barker’s and Brenner’s hypothesis; the latter, which supports the intrauterine origin of later-life health, explains the relationship between BW and the number of nephrons as well as the risk of developing hypertension and chronic kidney disease (CKD). Consequently, long-term cardiovascular and renal health disorders are likely to occur [7–9].

It has been evidenced that low birth weight (LBW), prematurity and growth restriction are markers of an adverse intrauterine environment whereas high birth weight (HBW), exposure to maternal diabetes and rapid growth during early childhood emerge as developmental risk factors for chronic diseases [10]. Compared with normal birth weight (NBW), LBW is associated with reduced kidney volume, increased risk of perinatal morbi-mortality, underweight and short stature. It is also linked to elevated blood pressure, hypertension, proteinuria, natriuresis, podocytopenia and focal segmental glomerulosclerosis, and occurrence and rapid progression of CKD [4, 5, 9–16]. Moreover, studies revealed that LBW is associated with male sex predilection of CKD and early onset of end stage renal disease in patients with autosomal dominant polycystic kidney disease, without racial predominance [12, 17–19]. In contrast, HBW increases the risk of hypertension in children and decreases the risk in adults; furthermore, it increases proteinuria in children with maternal diabetes as well as the risk of diabetes-associated end stage renal disease [10].

In sub-Saharan Africa, the relationship between BW and renal markers has been scarcely explored, especially in school-aged children. Studies assessing the relationship between BW and blood pressure revealed a consistently positive association in neonates, an inverse association among children and inconsistent results in adolescents [20–25]. LBW was also identified as a non-traditional risk factor for CKD in this setting, and contributed to the burden of disease [26].

In Cameroon, CKD is highly prevalent in adult populations, ranging from 10 to 14.2% and driven by known modifiable risk factors for chronic nephropathy [27, 28]. In children, studies revealed no association between BW and blood pressure (BP) [29, 30]. However, no study had yet assessed the spectrum of renal markers according to BW among children. In this context, we undertook the present study aiming to evaluate the relationship between BW and blood pressure, proteinuria and glomerular filtration rate (GFR) in children aged 5–10 years and living in Yaoundé, Cameroon.

## Methods

### Study design and setting

This was a cross-sectional hospital-based study carried out over a period of 4 months (January to April 2018) at the Yaounde Gynaeco-obstetric and Paediatric Hospital (YGOPH). The YGOPH is one of the tertiary health care facilities in Yaounde, the capital city of Cameroon. Inaugurated in 2002, the YGOPH has a capacity of 240 beds; it offers mainly gynaeco-obstetric and paediatric care to patients. The study was approved by the Institutional Review Board of the Faculty of Heath Sciences of the University of Buea and the Ethical Committee of the YGOPH.

### Study participants

The study involved children between 5 and 10 years, born between 2007 and 2012 at YGOPH, who used to consult in this hospital for pediatric problems, and whose parents/guardians gave their consent. We excluded from the study children suffering from renal and urinary tract malformations, CKD, diabetes, HIV infection, hepatitis B and C infection, and sickle cell disease. Children born between 2007 and 2012 were identified from the maternity registers and recorded in a book. Based on the BW of the maternity register, children were divided into 3 groups: LBW (< 2500 g), NBW (2500-3999 g) and HBW (≥4000 g). We used the sample size calculation formula for cross-sectional studies and considered the clinical prevalence of hypertension in children of 3.5% to obtain a minimum sample size of 70 participants [31, 32]; we selected 1 HBW child for 2 LBW and 4 NBW children. The recruitment of LBW and HBW participants was consecutive on their appearance in the register while NBW children were randomly selected. In case of refusal to participate, the next name on the register was selected and the procedure repeated.

### Data collection

For each eligible participant, the child’s parent/guardian was contacted through a phone call during which the study procedures were fully explained and an appointment fixed based on their availability. During the meeting with the parent/guardian and their child, the study was once again explained to them and an assent form signed. A self-designed and pre-tested questionnaire was used for data collection. Data collected included socio-demographic details (age, sex), clinical characteristics (weight, height, systolic and diastolic blood pressure), maternal history of pregnancy (age, type of pregnancy, maternal illness, smoking and alcohol consumption), birth characterisitics (weight, gestational term and age, and history of child reanimation and/or hospitalization) and laboratory parameters (proteinuria, and serum and urinary creatinine). We used an appropriate cuff size of 13.5 to 22 cm according to the American Academy of Pediatrics guidelines to measure BP [32]. After 5 min of rest with the participant in the sitting position, the back supported and feet uncrossed on the floor, we used an automated sphygmomanometer (OMRON HEM705CP, Omron Matsusaka Co, Matsusaka City, Mie-Ken, Japan) to measure BP on the right arm placed at the level of the heart, stretched out on the table with the palm facing up. The cuff was appropriately placed and then the machine was switched on. Three readings were taken consecutively and their mean calculated and recorded. Using BP table levels for sex, age and height percentiles, the BP percentile was recorded. When the BP was ≥90th percentile, it was repeated weekly up to two times; when it remained the same on the third measurement, it was measured using an aneroid sphygmomanometer twice consecutively; the mean of these measures were calculated and recorded as the final value.

Each participant provided 50 ml first morning mid-stream urine for an immediate semi-quantitative measurement of dipstick proteinuria using the CombiScreen 7SL PLUS 7 test strips (Analyticon Biotechnologies AG, D-35104 Lichentenfeis, Germany). Participants with at least traces on urine dipstick for proteinuria on the first sample were given an appointment 1 week later to repeat the urine dipstick. Those with a second urine sample still showing at least traces for proteinuria were seen 1 week later for another urine dipstick test. When the proteinuria persisted even just as traces after the second repeated urine dipstick, we proceeded to estimate the 24-h proteinuria from urine protein to creatinine ratio (PCR). We equally collected 3 ml of whole blood from an antecubital vein for serum creatinine and subsequent calculation of the GFR. Urine and blood samples were transported to the laboratory for processing. Serum and urinary creatinine were measured with a kinetic modification of the Jaffé reaction using a Human visual spectrophotometer (Human Gesellschaft, Biochemica und Diagnostica mbH, Wiesbaden, Germany) and Beckman creatinine analyzer (Beckman CX systems instruments, Anaheim, CA, USA) while urinary protein was measured using pyrogallol red-molybdate complex with Teco diagnostics tests (Teco Diagnostics, Anaheim, CA, USA).

### Definitions and calculations

Delivery was categorized as preterm (< 37 weeks of gestation), normal (37 to 42 weeks) or post-term (> 42 weeks). Small for gestational age (SGA) was defined by BW < 10th centile for that gestational age (GA) whereas large for GA (LGA) was a BW > 90th centile for that GA and appropriate for GA (AGA) corresponded to BW between the 10th and 90th centile for that GA. We grouped children in percentiles using the World Health Organisation height and weight for age percentile reference charts release in 2007 [33]. According to weight, underweight (<5th percentile for age), normal weight (5- < 95th percentile for age) or overweight (≥95th percentile for age) were distinguished. For height, short stature (< 5th percentile for age), normal height (5- < 95th percentile for age) or tall stature (≥95th percentile for age) were considered. BMI was estimated as weight (kg)/square height (m^2^). It was stratified into underweight (<5th percentile for age and sex), normal weight (5-85th percentile for age and sex), overweight (85-95th percentile for age and sex) and obesity (≥95th percentile for age and sex). BP was either normal [systolic blood pressure (SBP) and diastolic blood pressure (DBP)] <90th percentile for sex, age and height), elevated (SBP and/or DBP ≥90- < 95th percentile for sex, age and height) whereas hypertension was defined as SBP and/or DBP ≥ 95th percentile for sex, age and height after three occasions according to the American Academy of Pediatrics guidelines to define BP categories and stages [32]. Dipstick proteinuria was defined by a persistent proteinuria (at least traces) after three measurements. The 24 h proteinuria was estimated from PCR and proteinuria corresponded to a PCR ≥ 200 mg/g. The Schwartz equation was used for estimate glomerular filtration rate (eGFR) [34]; it was either increased (≥120 ml/min/1.73 m^2^), normal (90- < 120 ml/min/1.73 m^2^) or decreased (< 90 ml/min/1.73 m^2^).

### Statistical analysis

Data were entered and coded using EPI info version 7.0 and analysed using Statistical Package for Social Science (SPSS) version 23.0. Considering the non-Gaussian distribution of continuous variables, medians and interquartile ranges (IQR) were computed for continuous variables. Frequencies and proportions were computed for categorical variables. Frequencies were compared using the Fisher exact test or the Chi-square test where appropriate. To compare continuous variables according to BW strata, we used the non-parametric U-test of Mann-Whitney or the H-test of Kruskal-Wallis, where indicated. The Spearman correlation was used to correlate the BW with other continuous variables. A *p*-value was considered statistically significant at < 0.05.

## Results

### Sociodemographic and anthropometric characteristics of the study population

We included 80 children among whom 49 (61.2%) boys divided into 21 (26.2%) LBW, 45 (56.2%) NBW and 14 (15.5%) HBW. The median (IQR) age was 7.3 (6.3–8.1) years with no significant difference according to BW groups (*p* = 0.32). The median (IQR) for weight, height and BMI were respectively 23.3 (21.0–27.8) kg, 124 (116–134) cm and 15.3 (14.6–16.8) kg/m^2^ with no significant difference with respect to BW strata (all *p* > 0.211). There were 17 (21.2%) overweight/obese children without any difference with BW groups (*p* = 0.665), Table 1.

**Table 1:** Sociodemographic and anthropometric characteristics of the study population

Variables	Total	LBW	NBW	HBW	***P***-value
**N (%)**	80 (100)	21 (26.2)	45 (56.2)	14 (17.5)	–
**Age (years), median (IQR)**	7.3 (6.3–8.1)	6.9 (6.2–7.9)	7.1 (6.5–8.2)	7.2 (6.3–8.1)	0.7
**Boys, n (%)**	49 (61.2)	12 (57.1)	26 (57.8)	11 (78.6)	0.32
**Weight (kg), median (IQR)**	23.3 (21–27.8)	23 (20–25.3)	23 (21.5–26.7)	27.5 (20.6–32.8)	0.215
**Weight percentile, median (IQR)**	50 (15–82)	25 (15–62.5)	50 (15–80)	62.5 (24–90)	0.381
**Weight percentile groups, n (%)**					
< 5th	3 (3.8)	0 (0)	1 (2.2)	2 (14.3)	
5-94th	64 (80)	18 (85.7)	36 (80)	10 (71.4)	0.233
≥ 95th	13 (16.2)	3 (14.3)	8 (17.8)	2 (14.3)	
**Height (cm), median (IQR)**	124 (116–134)	123 (114–135)	122 (119–130)	128 (115–136)	0.577
**Height percentile, median (IQR)**	50.0 (25–82.5)	50 (20–75)	50 (20–85)	50 (25–78.8)	0.833
**Height percentile groups, n (%)**					
< 5th	2 (2.5)	1 (2.5)	1 (2.2)	0 (0)	
5-94th	70 (87.5)	18 (85.7)	39 (86.7)	13 (92,9)	0.862
≥ 95th	8 (10)	2 (9.5)	5 (11.1)	1 (7.1)	
**BMI (kg/m**^**2**^**), median (IQR)**	15.3 (14.6–16.8)	14.7 (14.1–16.8)	15.3 (14.8–17.1)	16.0 (14.8–17.5)	0.211
**BMI percentile, median (IQR)**	50 (25–75)	25 (10–75)	50 (25–75)	50 (22.5–86.3)	0.342
**BMI percentile groups, n (%)**					
< 5th	5 (6.2)	1 (4.8)	2 (4.4)	2 (14.3)	
5-84th	58 (72.5)	16 (76.2)	34 (75.6)	8 (57.1)	0.665
≥ 85th	17 (21.2)	4 (19)	9 (20)	4 (28.6)	

*BMI* Body mass index, *HBW* High birth weight, *IQR* Interquartile range, *LBW* Low birth weight, *NBW* Normal birth weight

### Maternal and birth history of participants

As presented in Table 2, the median (IQR) maternal age was 28 (23.2–32.0) years with no significant difference in BW (*p* = 0.486). Amongst the 10 (12.5%) of multiple pregnancies, 9 (90%) led to LBW infants with a statistical significance (*p* < 0.001). We observed that 3 (3.7%) women had diabetes mellitus among whom 2 (66.7%) delivered HBW children with a statistical significance (*p* = 0.048). In Table 3, the median (IQR) BW was 3200 (2421.5–3678.8) g with 2150 (1885–2375) g for LBW, 3200 (2900–3500) g for NBW and 4350 (4165–4642) g for HBW children (*p* < 0.001). The median (IQR) gestational term was 39 (38–40) weeks, with 10 (47.6%) LBW children born before 37 weeks (*p* < 0.001). All LBW children born before 37 weeks had a BW SGA whereas 1 (2.2%) NBW was LGA.

**Table 2:** Maternal history during pregnancy

Variables	Total	LBW	NBW	HBW	***P***-value
**N (%)**	80 (100)	21 (26.2)	45 (56.2)	14 (17.5)	–
**Maternal age (years), median (IQR)**	28 (23.2–32)	29 (24.5–33)	27 (23–31.5)	30 (24–34)	0.486
**Type of Pregnancy, n (%)**					
Single	70 (87.5)	12 (57.1)	44 (97.8)	14 (100)	
Multiple	10 (12.5)	9 (42.9)	1 (2.2)	0 (0)	< 0.001
**Maternal Illness, n (%)**					
Hypertension	4 (5.0)	2 (9.5)	2 (4.4)	0 (0)	0.335
Diabetes	3 (3.7)	1 (4.8)	0 (0)	2 (14.3)	0.048
HIV infection	3 (3.7)	1 (4.8)	1 (2.2)	1 (7.1)	0.687
Hepatitis B infection	1 (1.2)	0 (0)	1 (2.2)	0 (0)	0.09
**Smoking, n (%)**	1 (1.2)	0 (0)	1 (2.2)	0 (0)	0.5
**Alcohol consumption, n (%)**	12 (15)	3 (14.3)	8 (17.8)	1 (7.1)	0.582

*HBW* High birth weight, *IQR* Interquartile range, *LBW* Low birth weight, *NBW* Normal birth weight

**Table 3:** Birth Characteristics

Variables	Total	LBW	NBW	HBW	***P***-value
**N (%)**	80 (100.0)	21 (26.2)	45 (56.2)	14 (17.5)	–
**Birth weight (grams), median (IQR)**	3200 (2421.5–3678.8)	2150 (1885–2375)	3200 (2900–3500)	4350 (4165–4642)	< 0.001
**Gestational term (weeks), median (IQR)**	39 (38–40)	37 (34–38)	39 (38–40)	39.5 (38–40)	< 0.001
**Gestational term groups (weeks), n (%)**					
< 37	13 (16.2)	10 (47.6)	1 (2.2)	02 (14.3)	
37–42	67 (83.8)	11 (56.4)	44 (97.8)	12 (85.7)	< 0.001
> 42	0 (0)	0 (0)	0 (0)	0 (0)	
**Weight for gestational age, n (%)**					
SGA	21 (26.2)	21 (100)	0 (0)	0 (0)	
AGA	44 (55.0)	0 (0)	44 (97.8)	0 (0)	< 0.001
LGA	15 (18.8)	0 (0)	1 (2.2)	14 (100)	
**Child reanimated at birth, n (%)**	21 (26.2)	10 (47.6)	08 (17.8)	03 (21.4)	0.098
**Child hospitalised at birth, n (%)**	24 (30)	9 (42.9)	10 (22.2)	5 (35.7)	0.209

*AGA* Appropriate for gestational age, *HBW* High birth weight, *IQR* Interquartile range, *LBW* Low birth weight, *LGA* Large for gestational age, *NBW* Normal birth weight, *SGA* Small for gestational age

### Blood pressure characteristics

The median (IQR) SBP and DBP was respectively 91 (85.3–97.8) mmHg and 56 (52.0–58.8) mmHg, without any statistical significance according to BW groups (all *p* > 0.187). There were 2 (2.5%) children with an elevated BP; both had a NBW, giving a prevalence of 4.4% of NBW children with an elevated blood pressure. Hypertension was observed in 2 (2.5%) children who all had a LBW, giving a prevalence of 9.5% among LBW population (see Table 4). None of the HBW children had an elevated BP or hypertension. Hypertension and elevated BP were not significantly associated with BW (*p* = 0.233).

**Table 4:** Blood pressure characteristics

Variables	Total	LBW	NBW	HBW	***P***-value
**N (%)**	80 (100)	21 (26.2)	45 (56.2)	14 (17.5)	–
**SBP (mmHg), median (IQR)**	91 (85.3–97.8)	94 (85.5–100.5)	90 (85–95)	92 (89.3–101)	0.187
**SBP percentile, median (IQR)**	49 (49–50)	49 (49–51)	49 (49–49.5)	49 (49–51)	0.118
**DBP (mmHg), median (IQR)**	56 (52–58.8)	57 (53–60)	55 (51–57)	57 (50–60.3)	0.242
**DBP percentile, median (IQR)**	49 (49–50.1)	50 (49–51)	49 (49–50)	49.5 (49–50)	0.219
**SBP percentile groups, n (%)**					
< 90	78 (97.5)	19 (90.5)	45 (100)	14 (100)	
90–94	1 (1.2)	1 (4.8)	0 (0)	0 (0)	0.24
≥ 95	1 (1.2)	1 (4.8)	0 (0)	0 (0)	
**DBP percentile groups, n (%)**					
< 90	77 (96.2)	19 (97.5)	44 (97.8)	14 (100)	
90–94	2 (2.5)	1 (4.8)	1 (2.2)	0 ()	0.429
≥ 95	1 (1.2)	1 (4.8)	0 (0)	0 (0)	
**BP percentile groups, n (%)**					
< 90	76 (95)	19 (90.5)	43 (95.6)	14 (100)	
90–94	2 (2.5)	0 (0)	2 (4.4)	0 (0)	0.233
≥ 95	2 (2.5)	2 (9.5)	0 (0)	0 (0)	

*DBP* Diastolic blood pressure, *HBW* High birth weight, *IQR* Interquartile range, *LBW* Low birth weight, *NBW* Normal birth weight, *SBP* Systolic blood pressure

### Proteinuria and glomerular filtration rate characteristics

In Table 5, dipstick positive proteinuria was observed in 15 (18.8%) children, with a significantly higher prevalence in LBW (28.6%) and HBW (35.7%) children compared with NBW (8.1%) ones (*p* = 0.033). For the 15 children who performed PCR, the median (IQR) PCR was 185 (130–373) mg/g without any significant difference according to BW classes (*p* = 0.228). A proteinuria was noticed in 7 (8.7%) children with a higher prevalence in LBW (19%) and HBW (14.3%) in comparison to NBW (2.2%) children, at the limit of statistical significance (*p* = 0.051). The median (IQR) eGFR was 105.5 (90–118) ml/min/1.73m^2^ without any significant difference with regards to BW groups (*p* = 0.330). There was a decreased eGFR in 18 (22.5%) children with an increased prevalence according to BW groups, ranging from 3 (14.3%) LBW, 11 (24.4%) NBW to 4 (28.6%) HBW children (*p* = 0.818).

**Table 5:** Proteinuria and glomerular filtration rate characteristics

Variables	Total	LBW	NBW	HBW	***P***-value
**N (%)**	80 (100)	21 (26.2)	45 (56.2)	14 (17.5)	–
**Dipstick positive proteinuria, n (%)**	15 (18.8)	6 (28.6)	4 (8.1)	5 (35.7)	0.033
**PCR (mg/g), median (IQR) (*n*** **= 15)**	185 (130–373)	291 (162–455)	143 (96.5–215)	185 (85–215)	0.228
**SCr (mg/dl), median (IQR) (*n*** **= 80)**	0.64 (0.59–0.73)	0.62 (0.56–0.68)	0.66 (0.6–0.72)	0.63 (0.53–0.82)	0.339
**eGFR (ml/min/1.73m**^**2**^**), median (IQR) (*n*** **= 80)**	105.5 (90–118)	113 (95–124)	103 (89–114.5)	109 (87.2–122)	0.33
**PCR ratio (mg/g), n (%), (*n*** **= 15)**					
< 200	73 (91.2)	17 (81)	44 (97.8)	12 (85.7)	0.051
≥ 200	7 (8.7)	4 (19)	1 (2.2)	2 (14.3)	
**eGFR (ml/min/1.73m**^**2**^**), n (%) (*n*** **= 80)**					
≥ 120	18 (22.5)	6 (28.6)	9 (20)	3 (21.4)	
90–119	44 (55)	12 (57.1)	25 (55.6)	7 (50)	0.818
< 90	18 (22.5)	3 (14.3)	11 (24.4)	4 (28.6)	

*eGFR* Estimated glomerular filtration rate, *HBW* High birth weight, *IQR* Interquartile range, *LBW* Low birth weight, *NBW* Normal birth weight, *PCR* Protein to creatinine ratio, *SCr* Serum creatinine

### Effect of prematurity, weight for gestational age and overweight/obesity on blood pressure, proteinuria and glomerular filtration rate

As presented in Table 6, GA did not significantly affect any of these parameters. When comparing with NBW, LBW had significantly higher SBP (*p* = 0.029); however, LBW and HBW children had a trend toward an increase DBP, PCR and eGFR with no statistical significance. We observed that overweight/obese children had significantly high SBP and DBP, and a reduced eGFR (all *p* < 0.037).

**Table 6:** Effect of prematurity, birth weight, weight for gestational age and obesity on blood pressure, proteinuria and glomerular filtration rate

Variables	Gestational term			Birth weight				Weight for gestational age				Overweight/obesity		
	< 37 weeks	37–42 weeks	*p*	LBW	NBW	HBW	*p*	SGA	AGA	LGA	*p*	Yes	No	*p*
**SBP percentile**	49.0 (49.0–51.0)	49.0 (49.0–50.0)	0.264	49.0 (49.0–51.0)	49.0 (49.0–49.5)	49.0 (49.0–51.0)	0.118	49.0 (49.0–51.0)	49.0 (49.0–49.8)	49.0 (49.0–51.0)	0.134	50.0 (49.0–51.0)	49.0 (49.0–50.3)	0.018
**DBP percentile**	50.0 (49.0–51.0)	49.0 (49.0–50.0)	0.066	50.0 (49.0–51.0)	49.0 (49.0–50.0)	49.5 (49.0–50.0)	0.219	50.0 (49.0–51.0)	49.0 (49.0–50.0)	49.0 (49.0–50.0)	0.227	50.0 (49.0–51.0)	49.0 (49.0–50.0)	0.146
**SCr (mg/dl)**	0.64 (0.60–0.74)	0.64 (0.58–0.73)	0.695	0.63 (0.56–0.68)	0.66 (0.61–0.76)	0.64 (0.53–0.83)	0.334	0.63 (0.56–0.68)	0.66 (0.60–0.76)	0.64 (0.54–0.82)	0.368	0.69 (0.62–0.84)	0.63 (0.58–0.73)	0.058
**eGFR (ml/min)**	99.0 (91.0–117.5)	106.0 (90.0–118.0)	0.506	113.0 (95.0–124.0)	103.0 (89.5–114.5)	109.5 (87.3–122.0)	0.339	113.0 (95.0–124.0)	103.5 (89.3–115.3)	109.0 (88.0–117.0)	0.379	93.0 (81.5–109.5)	106.0 (93.0–121.0)	0.036

Estimates are median and interquartile range. *AGA* Appropriate for gestational age, *DBP* Diastolic blood pressure, *eGFR* Estimated glomerular filtration rate, *HBW* High birth weight, *LBW* Low birth weight, *LGA* Large for gestational age, *NBW* Normal birth weight, *SBP* Systolic blood pressure, *SCr* Serum creatinine, *SGA* Small for gestational age

### Correlations between birth weight and variables

There was an inverse relationship between BW and SBP, DBP, PCR and eGFR although with no significant difference (all *p* > 0.05). However, we observed a significant weak positive correlation between BW and weight (*r* = 0.231) as well as BMI (*r* = 0.269), and a negative correlation between BW and weight percentile (*r* = − 0.241) (all *p* < 0.039) (Table 7).

**Table 7:** Correlation between birth weight and others variables

Variables	Coefficient	***P***-value
**Maternal age**	0.096	0.397
**Weight**	0.231	0.039
**Weight percentile**	−0.241	0.031
**Height**	0.108	0.343
**Height percentile**	0.147	0.193
**BMI**	0.269	0.016
**BMI percentile**	0.240	0.032
**SBP**	−0.014	0.904
**SBP percentile**	−0.116	0.306
**DBP**	−0.004	0.975
**DBP percentile**	−0.078	0.493
**BP percentile**	−0.140	0.215
**PCR**	−0.181	0.520
**Serum creatinine**	0.106	0.350
**eGFR**	−0.073	0.518

*BMI* Body mass index, *BP* Blood pressure, *DBP* Diastolic blood pressure, *eGFR* Estimated glomerular filtration rate, *PCR* Protein to creatinine ratio, *SBP* Systolic blood pressure

## Discussion

This study revealed a high prevalence of elevated blood pressure and hypertension, observed respectively in NBW and LBW children only. Nearly one out of ten children had proteinuria which was significantly associated with LBW and HBW. More than one out of five children presented with a decreased eGFR associated with an increased prevalence with regards to BW. We noticed an inverse relationship between BW and renal markers (blood pressure, proteinuria and GFR), with no significant association.

The reported high prevalence of hypertension as well as an elevated blood pressure were previously observed in this setting [30]. We did not find any association between BW and BP overall as well as with SBP or DBP as reported earlier [29, 30]. However, we found that all children with hypertension were of the LBW group, which could be explained by the Brenner’s hypothesis. Indeed, it supports a relationship between BW and the number of nephrons as well as the risk of developing hypertension [8, 20, 21]. Regarding the presence of hypertension in only LBW children, there is need for regular BP follow-up in this group of children.

Further, we observed that LBW and HBW children had significant proteinuria compared to NBW children, as reported elsewhere [10, 12]. This could be related to hyperfiltration mechanisms related either to reduced glomerular number in LBW infants, the association between HBW and maternal diabetes or glomerular lesions such as podocytopenia and focal segmental glomerulosclerosis [10].

There was no significant association between eGFR and BW as reported elsewhere in children with similar age using creatinine [19, 35, 36]. However, the eGFR using cystatin C showed an inverse relationship with BW [36]. This could be explained by the fact that cystatin C reflects better the eGFR in comparison to creatinine and suggests its preferential use in LBW children. Nevertheless, higher GFRs turned to be more frequent in the LBW children compared to NBW and HBW. This could be explained by the fact that LBW is associated with a reduced number of nephrons and a low glomerular density at birth; glomerular enlargement volume, hyperfiltration, increased GFR and proteinuria occur during childhood and adolescence due compensatory maladaptive changes as consequences of compromised nephrogenesis; furthermore with advanced age favored by overweight/obesity, there will be a reduction in the GFR and worsening of proteinuria due to focal segemental glomerulosclerosis lesions [1–3, 37].

We observed an inverse relationship between BW and renal markers without any statistical significance as previously observed [21, 29]. However, studies in SSA reported a significant inverse relationship between BW and BP [20, 25]. Meanwhile, some other studies showed a positive correlation between BW and SBP at birth [24]. The difference of correlation between renal markers and BW observed in this study compared to others could be related to the difference in the age of participants and the method of renal markers measurement.

### Strengths and limitations

The main limitations of this study were the estimation of GFR with creatinine instead of cystatin C which reflects better, the kidney function. Furthermore, the small sample size as well as the number of events might have an influence on possible lack of associations. Nevertheless, this study is the first in Central Africa, to the very best of our knowledge, to assess the relationship between BW and overall renal markers in childhood. We used up-to-date guidelines for blood pressure and proteinuria diagnosis in children [32, 38]. These findings contribute to enrich data on renal markers and BW in the sub-Saharan Africa setting and suggest further research using cystatin C-based equations for eGFR.

## Conclusion

The present study shows that childhood life with history of abnormal birth weight can be impacted by foetal life; thus affecting BP and urine protein excretion rate. This study highlighted the importance for regular follow-up of children with LBW and HBW in order to implement early nephroprotective measures. Therefore, lifestyle education is a key; it can begin to be delivered to mothers of at risk babies identified in the delivery room and during the baby and maternal follow-up. We also suggest the implementation of annual screening of at risk children, overweight or obese, in order to identify hypertension, proteinuria and reduced eGFR.

## Data Availability

Data and materials are available with corresponding author which is the principal investigator. They can be consulted at anytime upon request. However, the ethical clearance and the inform consent form did mention that patient data could be share to a third party.

## Abbreviations

AGA: Appropriate for gestational age
BMI: Body mass index
BP: Blood pressure
DBP: Diastolic blood pressure
eGFR: Estimated glomerular filtration rate
HBW: High birth weight
GA: Gestational age
IQR: Interquartile range
LGA: Large for gestational age
LBW: Low birth weight
NBW: Normal birth weight
PCR: Proteinto creatinine ratio
SBP: Systolic blood pressure
SCr: Serum creatinine
SGA: Small for gestational age
